# Learning local equivariant representations for large-scale atomistic dynamics

**DOI:** 10.1038/s41467-023-36329-y

**Published:** 2023-02-03

**Authors:** Albert Musaelian, Simon Batzner, Anders Johansson, Lixin Sun, Cameron J. Owen, Mordechai Kornbluth, Boris Kozinsky

**Affiliations:** 1 Harvard University, Cambridge, MA USA; 2 Robert Bosch LLC Research and Technology Center, Cambridge, MA USA

**Keywords:** Atomistic models, Computational science, Computational methods

## Abstract

A simultaneously accurate and computationally efficient parametrization of the potential energy surface of molecules and materials is a long-standing goal in the natural sciences. While atom-centered message passing neural networks (MPNNs) have shown remarkable accuracy, their information propagation has limited the accessible length-scales. Local methods, conversely, scale to large simulations but have suffered from inferior accuracy. This work introduces Allegro, a strictly local equivariant deep neural network interatomic potential architecture that simultaneously exhibits excellent accuracy and scalability. Allegro represents a many-body potential using iterated tensor products of learned equivariant representations without atom-centered message passing. Allegro obtains improvements over state-of-the-art methods on QM9 and revMD17. A single tensor product layer outperforms existing deep MPNNs and transformers on QM9. Furthermore, Allegro displays remarkable generalization to out-of-distribution data. Molecular simulations using Allegro recover structural and kinetic properties of an amorphous electrolyte in excellent agreement with ab-initio simulations. Finally, we demonstrate parallelization with a simulation of 100 million atoms.

## Abstract (web summary)

The paper presents a method that allows scaling machine learning interatomic potentials to extremely large systems, while at the same time retaining the remarkable accuracy and learning efficiency of deep equivariant models. This is obtained with an E(3)- equivariant neural network architecture that combines the high accuracy of equivariant neural networks with the scalability of local methods.

## Introduction

Molecular dynamics (MD) and Monte-Carlo (MC) simulation methods are a core pillar of computational chemistry, materials science, and biology. Common to a diverse set of applications ranging from energy materials^1^ to protein folding^2^ is the requirement that predictions of the potential energy and atomic forces must be both accurate and computationally efficient to faithfully describe the evolution of complex systems over long timescales. While first-principles methods such as density functional theory (DFT), which explicitly treat the electrons of the system, provide an accurate and transferable description of the system, they exhibit poor scaling with system size and thus limit practical applications to small systems and short simulation times. Classical force fields based on simple functions of atomic coordinates are able to scale to large systems and long timescales but are inherently limited in their fidelity and can yield unfaithful dynamics. Descriptions of the potential energy surface (PES) using machine learning (ML) have emerged as a promising approach to move past this trade-off^3–24^. Machine learning interatomic potentials (MLIPs) aim to approximate a set of high-fidelity energy and force labels with improved computational efficiency that scales linearly in the number of atoms. A variety of approaches have been proposed, from shallow neural networks and kernel-based approaches^3–6^ to more recent methods based on deep learning^14,15,20,25,26^. In particular, a class of MLIPs based on atom-centered message-passing neural networks (MPNNs) has shown remarkable accuracy^9,11,14,15,26,27^. In interatomic potentials based on MPNNs, an atomistic graph is induced by connecting each atom (node) to all neighboring atoms inside a finite cutoff sphere surrounding it. Information is then iteratively propagated along this graph, allowing MPNNs to learn many-body correlations and access non-local information outside of the local cutoff. This iterated propagation, however, leads to large receptive fields with many effective neighbors for each atom, which impedes parallel computation and limits the length scales accessible to atom-centered message-passing MLIPs. MLIPs using strictly local descriptors such as Behler-Parrinello neural networks^5^, GAP^6^, SNAP^7^, DeepMD^20^, Moment Tensor Potentials^8^, or ACE^12^ do not suffer from this obstacle due to their strict locality. As a result, they can be easily parallelized across devices and have been successfully scaled to extremely large system sizes^28–31^. Approaches based on local descriptors, however, have so far fallen behind in accuracy compared to state-of-the-art equivariant, atom-centered message passing interatomic potentials^15^.

### Message-passing interatomic potentials

Message-passing neural networks (MPNNs) which learn atomistic representations have recently gained popularity in atomistic machine learning due to advantages in accuracy compared to hand-crafted descriptors. Atom-centered message-passing interatomic potentials operate on an atomistic graph constructed by representing atoms as nodes and defining edges between atoms that are within a fixed cutoff distance of one another. Each node is then represented by a hidden state ${{{{{{{{\bf{h}}}}}}}}}_{i}^{t}\in {{\mathbb{R}}}^{c}$ representing the state of atom *i* at layer *t*, and edges are represented by edge features **e**_*ij*_, for which the interatomic distance *r*_*ij*_ is often used. The message-passing formalism can then be concisely described as^32^:

1 $${{{{{{{{\bf{m}}}}}}}}}_{i}^{t+1}=\mathop{\sum}\limits_{j\in {{{{{{{\mathcal{N}}}}}}}}(i)}{M}_{t}\left({{{{{{{{\bf{h}}}}}}}}}_{i}^{t},{{{{{{{{\bf{h}}}}}}}}}_{j}^{t},{{{{{{{{\bf{e}}}}}}}}}_{ij}\right)$$

2 $${{{{{{{{\bf{h}}}}}}}}}_{i}^{t+1}={U}_{t}\left({{{{{{{{\bf{h}}}}}}}}}_{i}^{t},{{{{{{{{\bf{m}}}}}}}}}_{i}^{t+1}\right)$$

where *M*_*t*_ and *U*_*t*_ are an arbitrary message function and node update function, respectively. From this propagation mechanism, it is immediately apparent that as messages are communicated over a sequence of *t* steps, the local receptive field of an atom *i*, i.e., the effective set of neighbors that contribute to the final state of atom *i*, grows approximately cubically with the effective cutoff radius *r*_*c*,*e*_. In particular, given a MPNN with *N*_layer_ message-passing steps and local cutoff radius of *r*_*c*,*l*_, the effective cutoff is *r*_*c*,*e*_ = *N*_layer_*r*_*c*,*l*_. Information from all atoms inside this receptive field feeds into a central atom’s state **h**_*i*_ at the final layer of the network. Due to the cubic growth of the number of atoms inside the receptive field cutoff *r*_*c*,*e*_, parallel computation can quickly become unmanageable, especially for extended periodic systems. As an illustrative example, we may take a structure of 64 molecules of liquid water at pressure *P* = 1 bar and temperature *T* = 300 K. For a typical setting of *N*_*t*_ = 6 message-passing layers with a local cutoff of *r*_*c*,*l*_ = 6 Å this would result in an effective cutoff of *r*_*c*,*e*_ = 36 Å. While each atom only has approximately 96 atoms in its local 6 Å environment (including the central atom), it has 20,834 atoms inside the extended 36 Å environment. Due to the atom-centered message-passing mechanism, information from each of these atoms flows into the current central atom. In a parallel scheme, each worker must have access to the high-dimensional feature vectors **h**_*i*_ of all 20,834 nodes, while the strictly local scheme only needs to have access to approximately 6^3^ = 216 times fewer atoms’ states. From this simple example, it becomes obvious that massive improvements in scalability can be obtained from strict locality in machine learning interatomic potentials. It should be noted that conventional, atom-centered message passing allows for the possibility, in principle, to capture long-range interactions (up to *r*_*c*,*e*_) and can induce many-body correlations. The relative importance of these effects in describing molecules and materials is an open question, and one of the aims of this work is to explore whether many-body interactions can be efficiently captured without increasing the effective cutoff.

### Equivariant neural networks

The physics of atomic systems is unchanged under the action of a number of geometric symmetries—rotation, inversion, and translation—which together comprise the Euclidean group *E*(3) (rotation alone is *SO*(3), and rotation and inversion together comprise *O*(3)). Scalar quantities such as the potential energy are invariant to these symmetry group operations, while vector quantities such as the atomic forces are equivariant to them and transform correspondingly when the atomic geometry is transformed. More formally, a function between vector spaces *f* : *X* → *Y* is equivariant to a group *G* if

3 $$f({D}_{X}[g]x)={D}_{Y}[g]f(x)\quad \forall g\in G,\forall x\in X$$

where *D*_*X*_[*g*] ∈ *GL*(*X*) is the representation of the group element *g* in the vector space *X*. The function *f* is invariant if *D*_*Y*_[*g*] is the identity operator on *Y*: in this case, the output is unchanged by the action of symmetry operations on the input *x*.

Most existing MLIPs guarantee the invariance of their predicted energies by acting only on invariant inputs. In invariant, atom-centered message-passing interatomic potentials in particular, each atom’s hidden latent space is a feature vector consisting solely of invariant scalars^25^. More recently, however, a class of models known as equivariant neural networks^33–36^ have been developed which can act directly on non-invariant geometric inputs, such as displacement vectors, in a symmetry-respecting way. This is achieved by using only *E*(3)-equivariant operations, yielding a model whose internal features are equivariant with respect to the 3D Euclidean group. Building on these concepts, equivariant architectures have been explored for developing interatomic potential models. Notably, the NequIP model^15^, followed by several other equivariant implementations^26,27,37–39^, demonstrated unprecedentedly low error on a large range of molecular and materials systems, accurately describes structural and kinetic properties of complex materials, and exhibits remarkable sample efficiency. In both the present work and in NequIP, the representation *D*_*X*_[*g*] of an operation *g* ∈ *O*(3) on an internal feature space *X* takes the form of a direct sum of irreducible representations (commonly referred to as irreps) of *O*(3). This means that the feature vectors themselves are comprised of various geometric tensors corresponding to different irreps that describe how they transform under symmetry operations. The irreps of *O*(3), and thus the features, are indexed by a rotation order *ℓ* ≥ 0 and a parity *p* ∈ (−1, 1). A tensor that transforms according to the irrep *ℓ*, *p* is said to “inhabit” that irrep. We note that in many cases one may also omit the parity index to instead construct features that are only *SE*(3)-equivariant (translation and rotation), which simplifies the construction of the network and reduces the memory requirements.

A key operation in such equivariant networks is the tensor product of representations, an equivariant operation that combines two tensors **x** and **y** with irreps *ℓ*_1_, *p*_1_ and *ℓ*_2_, *p*_2_ to give an output inhabiting an irrep *ℓ*_out_, *p*_out_ satisfying ∣*ℓ*_1_ − *ℓ*_2_∣ ≤ *ℓ*_out _≤ ∣*ℓ*_1_ + *ℓ*_2_∣ and *p*_out_ = *p*_1_*p*_2_:

4 $${({{{{{{{\bf{x}}}}}}}}\otimes {{{{{{{\bf{y}}}}}}}})}_{{\ell }_{{{{{{{{\rm{out}}}}}}}}},{m}_{{{{{{{{\rm{out}}}}}}}}}}=\mathop{\sum}\limits_{{m}_{1},{m}_{2}}\left(\begin{array}{ccc}{\ell }_{1}&{\ell }_{2}&{\ell }_{{{{{{{{\rm{out}}}}}}}}}\\ {m}_{1}&{m}_{2}&{m}_{{{{{{{{\rm{out}}}}}}}}}\end{array}\right){{{{{{{{\bf{x}}}}}}}}}_{{\ell }_{1},{m}_{1}}{{{{{{{{\bf{y}}}}}}}}}_{{\ell }_{2},{m}_{2}}$$

where $$\left(\begin{array}{rcl}{\ell }_{1}&{\ell }_{2}&{\ell }_{{{{{{{{\rm{out}}}}}}}}}\\ {m}_{1}&{m}_{2}&{m}_{{{{{{{{\rm{out}}}}}}}}}\end{array}\right)$$ is the Wigner 3*j* symbol. Two key properties of the tensor product are that it is bilinear (linear in both **x** and **y**) and that it combines tensors inhabiting different irreps in a symmetrically valid way. Many simple operations are encompassed by the tensor product, such as for example:

- scalar-scalar multiplication: (*ℓ*_1_ = 0, *p*_1_ = 1), (*ℓ*_2_ = 0, *p*_2_ = 1) → (*ℓ*_out_ = 0, *p*_out_ = 1)
- vector dot product: (*ℓ*_1_ = 1, *p*_1_ = −1), (*ℓ*_2_ = 1, *p*_2_ = − 1) → (*ℓ*_out_ = 0, *p*_out_ = 1)
- vector cross product, resulting in a pseudovector: (*ℓ*_1_ = 1, *p*_1_ = −1), (*ℓ*_2_ = 1, *p*_2_ = − 1) → (*ℓ*_out_ = 1, *p*_out_ = 1).

The message function ${M}_{t}({{{{{{{{\bf{h}}}}}}}}}_{i}^{t},{{{{{{{{\bf{h}}}}}}}}}_{j}^{t},{{{{{{{{\bf{e}}}}}}}}}_{ij})$ of the NequIP model, for example, uses this tensor product to define a message from atom *j* to *i* as a tensor product between equivariant features of the edge *ij* and the equivariant features of the neighboring node *j*.

### Atomic cluster expansion

Finally, parallel to atom-centered message-passing interatomic potentials, the Atomic Cluster Expansion (ACE) has been developed as a unifying framework for various descriptor-based MLIPs^12^. ACE can also be expressed in terms of the same tensor product operation introduced above, with further details provided in “Methods”.

In this work, we present Allegro, an equivariant deep-learning approach that retains the high accuracy of the recently proposed class of equivariant MPNNs^15,26,27,37,39,40^ while combining it with strict locality and thus the ability to scale to large systems. We demonstrate that Allegro not only obtains state-of-the-art accuracy on a series of different benchmarks but can also be parallelized across devices to access simulations with hundreds of millions of atoms. We further find that Allegro displays a high level of transferability to out-of-distribution data, significantly outperforming other local MLIPs, in particular including body-ordered approaches. Finally, we show that Allegro can faithfully recover structural and kinetic properties from molecular dynamics simulations of Li_3_PO_4_, a complex phosphate electrolyte.

The outline of the article is as follows: we first surveyed relevant related work on message-passing interatomic potentials, equivariant neural networks, and the atomic cluster expansion. We then outline the core ideas and design of the Allegro approach, followed by a series of results on standard benchmarks. Finally, we show the results of molecular dynamics simulations on a challenging material, an analysis of the scaling properties of Allegro, and a theoretical analysis of the framework.

## Results

In the following, we describe the proposed method for learning high-dimensional potential energy surfaces using strictly local many-body equivariant representations.

### Energy decomposition

We start by decomposing the potential energy of a system into per-atom energies *E*_*i*_, in line with previous approaches^5,6,25^:

5 $${E}_{{{{{{{{\rm{system}}}}}}}}}=\mathop{\sum }\limits_{i}^{N}{\sigma }_{{Z}_{i}}{E}_{i}+{\mu }_{{Z}_{i}}$$

where ${\sigma }_{{Z}_{i}}$ and ${\mu }_{{Z}_{i}}$ are per-species scale and shift parameters, which may be trainable. Unlike most existing MLIPs, we further decompose the per-atom energy into a sum of pairwise energies, indexed by the central atom and one of its local neighbors

6 $${E}_{i}=\mathop{\sum}\limits_{j\in {{{{{{{\mathcal{N}}}}}}}}(i)}{\sigma }_{{Z}_{i},{Z}_{j}}{E}_{ij}$$

where *j* ranges over the neighbors of atom *i*, and again one may optionally apply a per-species-pair scaling factor ${\sigma }_{{Z}_{i},{Z}_{j}}$. It is important to note that while these pairwise energies are indexed by the atom *i* and its neighbor *j*, they may depend on all neighboring atoms *k* belonging to the local environment ${{{{{{{\mathcal{N}}}}}}}}(i)$. Because *E*_*ij*_ and *E*_*ji*_ contribute to different site energies *E*_*i*_ and *E*_*j*_, respectively, we choose that they depend only on the environments of the corresponding central atoms. As a result and by design, *E*_*ij*_ ≠ *E*_*ji*_. Finally, the force acting on atom *i*, ${\overrightarrow{F}}_{i}$, is computed using autodifferentiation according to its definition as the negative gradient of the total energy with respect to the position of atom *i*:

$${\overrightarrow{F}}_{i}=-{\nabla }_{i}{E}_{{{{{{{{\rm{system}}}}}}}}}$$

which gives an energy-conserving force field.

### The Allegro model

**Fig. 1:**
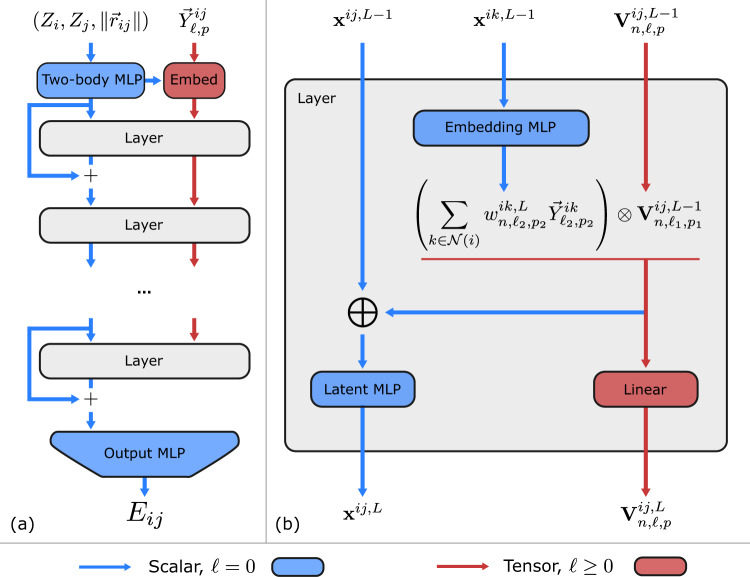
The Allegro network. **a** shows the Allegro model architecture and **b** details a tensor product layer. Blue and red arrows represent scalar and tensor information, respectively, ⊗ denotes the tensor product, and ⊕ is concatenation.

The Allegro architecture, shown in Fig. 1, is an arbitrarily deep equivariant neural network with *N*_layer_ ≥ 1 layers. The architecture learns representations associated with ordered pairs of neighboring atoms using two latent spaces: an invariant latent space, which consists of scalar (*ℓ* = 0) features, and an equivariant latent space, which processes tensors of arbitrary rank *ℓ* ≥ 0. The two latent spaces interact with each other at every layer. The final pair energy *E*_*ij*_ is then computed by a multi-layer perceptron (MLP) acting on the final layer’s scalar features.

We use the following notations:

- ${\overrightarrow{r}}_{i}$: position of the *i*th atom in the system
- ${\overrightarrow{r}}_{ij}$: relative displacement vector ${\overrightarrow{r}}_{j}-{\overrightarrow{r}}_{i}$ from *i* to *j*
- *r*_*ij*_: corresponding interatomic distance
- ${\hat{r}}_{ij}$: unit vector of ${\overrightarrow{r}}_{ij}$
- ${\overrightarrow{Y}}_{\ell,p}^{ij}$: projection of ${\hat{r}}_{ij}$ onto the *ℓ*-th real spherical harmonic which has parity *p* = (−1)^*ℓ*^. We omit the *m* = − *ℓ*, . . . , 0, . . . *ℓ* index within the representation from the notation for compactness
- *Z*_*i*_: chemical species of atom *i*
- MLP(. . . ): multi-layer perceptron—a fully connected scalar neural network, possibly with nonlinearities
- **x**^*ij*,*L*^: invariant scalar latent features of the ordered pair of atoms *ij* at layer *L*
- ${{{{{{{{\bf{V}}}}}}}}}_{n,\ell,p}^{ij,L}$: equivariant latent features of the ordered pair of atoms *ij* at layer *L*. These transform according to a direct sum of irreps indexed by the rotation order *ℓ* ∈ 0, 1, . . . , *ℓ*_max_ and parity *p* ∈ − 1, 1 and thus consist of both scalars (*ℓ* = 0) and higher-order tensors (*ℓ* > 0). The hyperparameter *ℓ*_max_ controls the maximum rotation order to which features in the network are truncated. In Allegro, *n* denotes the channel index which runs over 0, . . . , *n*_equivariant_ − 1. We omit the *m* index within each irreducible representation from the notation for compactness.

#### Two-body latent embedding

Before the first tensor product layer, the scalar properties of the pair *ij* are embedded through a nonlinear MLP to give the initial scalar latent features **x**^*ij*,*L*=0^:

7 $${{{{{{{{\bf{x}}}}}}}}}^{ij,L=0}={{{{{{{{\rm{MLP}}}}}}}}}_{{{{{{{{\rm{two-body}}}}}}}}}\left({{{{{{{\rm{1Hot}}}}}}}}({Z}_{i})\parallel {{{{{{{\rm{1Hot}}}}}}}}({Z}_{j})\parallel B({r}_{ij})\right)\cdot u({r}_{ij})$$

where ∥ denotes concatenation, 1Hot( ⋅ ) is a one-hot encoding of the center and neighbor atom species *Z*_*i*_ and *Z*_*j*_, and

8 $$B({r}_{ij})=({B}_{1}({r}_{ij})\parallel ...\parallel {B}_{{N}_{{{{{{{{\rm{basis}}}}}}}}}}({r}_{ij}))\left.\right)$$

is the projection of the interatomic distance *r*_*ij*_ onto a radial basis. We use the Bessel basis functions with a polynomial envelope function as proposed in ref. ^14^. The basis is normalized as described in Supplementary Note 1 and plotted in Supplementary Fig. 1. Finally, the function $u({r}_{ij}):{\mathbb{R}}\to {\mathbb{R}}$ by which the output of MLP_two-body_ is multiplied is the same smooth cutoff envelope function as used in the radial basis function.

The initial equivariant features ${{{{{{{{\bf{V}}}}}}}}}_{n,\ell,p}^{ij,L=0}$ are computed as a linear embedding of the spherical harmonic projection of ${\hat{r}}_{ij}$:

9 $${{{{{{{{\bf{V}}}}}}}}}_{n,\ell,p}^{ij,L=0}={w}_{n,\ell,p}^{ij,L=0}{\overrightarrow{Y}}_{\ell,p}^{ij}$$

where the channel index is *n* = 0, . . . , *n*_equivariant_ − 1, and where the scalar weights ${w}_{n,\ell,p}^{ij,L=0}$ for each center-neighbor pair *ij* are computed from the initial two-body scalar latent features:

10 $${w}_{n,\ell,p}^{ij,L=0}={{{{{{{{\rm{MLP}}}}}}}}}_{{{{{{{{\rm{embed}}}}}}}}}^{L=0}{({{{{{{{{\bf{x}}}}}}}}}^{ij,L=0})}_{n,\ell,p}.$$

#### Layer architecture

Each Allegro tensor product layer consists of four components:

1. an MLP that generates weights to embed the central atom’s environment
2. an equivariant tensor product using those weights
3. an MLP to update the scalar latent space with scalar information resulting from the tensor product
4. an equivariant linear layer that mixes channels in the equivariant latent space.

*Tensor product*: Our goal is to incorporate interactions between the current equivariant state of the center-neighbor pair and other neighbors in the environment, and the most natural operation with which to interact equivariant features is the tensor product. We thus define the updated equivariant features on the pair *ij* as a weighted sum of the tensor products of the current features with the geometry of the various other neighbor pairs *ik* in the local environment of *i*:

11 $${{{{{{{{\bf{V}}}}}}}}}_{n,({\ell }_{1},{p}_{1},{\ell }_{2},{p}_{2})\to ({\ell }_{{{{{{{{\rm{out}}}}}}}}},{p}_{{{{{{{{\rm{out}}}}}}}}})}^{ij,L}=\mathop{\sum}\limits_{k\in {{{{{{{\mathcal{N}}}}}}}}(i)}{w}_{n,{\ell }_{2},{p}_{2}}^{ik,L}\left({{{{{{{{\bf{V}}}}}}}}}_{n,{\ell }_{1},{p}_{1}}^{ij,L-1}\otimes {\overrightarrow{Y}}_{{\ell }_{2},{p}_{2}}^{ik}\right)$$

12 $$=\mathop{\sum}\limits_{k\in {{{{{{{\mathcal{N}}}}}}}}(i)}{{{{{{{{\bf{V}}}}}}}}}_{n,{\ell }_{1},{p}_{1}}^{ij,L-1}\otimes \left({w}_{n,{\ell }_{2},{p}_{2}}^{ik,L}{\overrightarrow{Y}}_{{\ell }_{2},{p}_{2}}^{ik}\right)$$

13 $$={{{{{{{{\bf{V}}}}}}}}}_{n,{\ell }_{1},{p}_{1}}^{ij,L-1}\otimes \left(\mathop{\sum}\limits_{k\in {{{{{{{\mathcal{N}}}}}}}}(i)}{w}_{n,{\ell }_{2},{p}_{2}}^{ik,L}{\overrightarrow{Y}}_{{\ell }_{2},{p}_{2}}^{ik}\right)$$

In the second and third lines, we exploit the bilinearity of the tensor product in order to express the update in terms of one tensor product, rather than one for each neighbor *k*, which saves significant computational effort. This is a variation on the “density trick”^6,41^.

We note that valid tensor product paths are all those satisfying ∣*ℓ*_1_ − *ℓ*_2_∣ ≤ *ℓ*_out_ ≤ ∣*ℓ*_1_ + *ℓ*_2_∣ and *p*_out_ = *p*_1_*p*_2_, so it is possible to have (*ℓ*_1_, *p*_1_) ≠ (*ℓ*_2_, *p*_2_) ≠ (*ℓ*_out_, *p*_out_). We additionally enforce ${\ell }_{{{{{{{{\rm{out}}}}}}}}}\le {\ell }_{\max }$. Which tensor product paths to include is a hyperparameter choice. In this work we include all allowable paths but other choices, such as restricting (*ℓ*_out_, *p*_out_) to be among the values of (*ℓ*_1_, *p*_1_), are possible.

*Environment embedding:* The second argument to the tensor product, ${\sum }_{k\in {{{{{{{\mathcal{N}}}}}}}}(i)}{w}_{n,{\ell }_{2},{p}_{2}}^{ik,L}{\overrightarrow{Y}}_{{\ell }_{2},{p}_{2}}^{ik}$, is a weighted sum of the spherical harmonic projections of the various neighbor atoms in the local environment. This can be viewed as a weighted spherical harmonic basis projection of the atomic density, similar to the projection onto a spherical-radial basis used in ACE^12^ and SOAP^41^. For this reason, we refer to ${\sum }_{k\in {{{{{{{\mathcal{N}}}}}}}}(i)}{w}_{n,{\ell }_{2},{p}_{2}}^{ik,L}{\overrightarrow{Y}}_{{\ell }_{2},{p}_{2}}^{ik}$ as the “embedded environment” of atom *i*.

A central difference from the atomic density projections used in descriptor methods, however, is that the weights of the sum are learned. In descriptor approaches such as ACE, the *n* index runs over a pre-determined set of radial–chemical basis functions, which means that the size of the basis must increase with both the number of species and the desired radial resolution. In Allegro, we instead leverage the previously learned scalar featurization of each center-neighbor pair to further learn

14 $${w}_{n,{\ell }_{2},{p}_{2}}^{ik,L}={{{{{{{{\rm{MLP}}}}}}}}}_{{{{{{{{\rm{embed}}}}}}}}}^{L}{({{{{{{{{\bf{x}}}}}}}}}^{ik,L-1})}_{n,{\ell }_{2},{p}_{2}}$$

which yields an embedded environment with a fixed, chosen number of channels *n*_equivariant_. It is important to note that ${w}_{n,{\ell }_{2},{p}_{2}}^{ik,L}$ is learned as a function of the existing scalar latent representation of the center-neighbor pair *ik* from previous layers. At later layers, this can contain significantly more information about the environment of *i* than a two-body radial basis. We typically choose MLP_embed_ to be a simple one-layer linear projection of the scalar latent space.

*Latent MLP*: Following the tensor product defined in Eq. (11), the scalar outputs of the tensor product are reintroduced into the scalar latent space as follows:

15 $${{{{{{{{\bf{x}}}}}}}}}^{ij,L}={{{{{{{{\rm{MLP}}}}}}}}}_{{{{{{{{\rm{latent}}}}}}}}}^{L}\left({{{{{{{{\bf{x}}}}}}}}}^{ij,L-1}\parallel \mathop{\bigoplus}\limits_{({\ell }_{1},{p}_{1},{\ell }_{2},{p}_{2})}{{{{{{{{\bf{V}}}}}}}}}_{n,({\ell }_{1},{p}_{1},{\ell }_{2},{p}_{2})\to ({\ell }_{{{{{{{{\rm{out}}}}}}}}}=0,{p}_{{{{{{{{\rm{out}}}}}}}}}=1)}^{ij,L}\right)\cdot u({r}_{ij})$$

where ∥ denotes concatenation and ⊕ denotes concatenation over all tensor product paths whose outputs are scalars (*ℓ*_out_ = 0, *p*_out_ = 1), each of which contributes *n*_equivariant_ scalars. The function $u({r}_{ij}):{\mathbb{R}}\to {\mathbb{R}}$ is again the smooth cutoff envelope from Eq. (7). The purpose of the latent MLP is to compress and integrate information from the tensor product, whatever its dimension, into the fixed dimension invariant latent space. This operation completes the coupling of the scalar and equivariant latent spaces since the scalars taken from the tensor product contain information about non-scalars previously only available to the equivariant latent space.

*Mixing equivariant features:* Finally, the outputs of various tensor product paths with the same irrep (*ℓ*_out_, *p*_out_) are linearly mixed to generate output equivariant features ${{{{{{{{\bf{V}}}}}}}}}_{n,\ell,p}^{ij,L}$ with the same number of channels indexed by *n* as the input features had:

16 $${{{{{{{{\bf{V}}}}}}}}}_{n,\ell,p}^{ij,L}=\mathop{\sum}\limits_{\begin{array}{c}{n}^{{\prime} }\\ ({\ell }_{1},{p}_{1},{\ell }_{2},{p}_{2})\end{array}}{w}_{n,{n}^{{\prime} },({\ell }_{1},{p}_{1},{\ell }_{2},{p}_{2})\to (\ell,p)}^{L}{{{{{{{{\bf{V}}}}}}}}}_{{n}^{{\prime} },({\ell }_{1},{p}_{1},{\ell }_{2},{p}_{2})\to (\ell,p)}^{ij,L}.$$

The weights ${w}_{n,{n}^{{\prime} },({\ell }_{1},{p}_{1},{\ell }_{2},{p}_{2})\to (\ell,p)}^{L}$ are learned. This operation compresses the equivariant information from various paths with the same output irrep (*ℓ*, *p*) into a single output space regardless of the number of paths.

We finally note that an *SE*(3)-equivariant version of Allegro, which is sometimes useful for computational efficiency, can be constructed identically to the *E*(3)-equivariant model described here by simply omitting all parity subscripts *p*.

#### Residual update

After each layer, Allegro uses a residual update^42^ in the scalar latent space that updates the previous scalar features from layer *L* − 1 by adding the new features to them (see Supplementary Note 2). The residual update allows the network to easily propagate scalar information from earlier layers forward.

#### Output block

To predict the pair energy *E*_*ij*_, we apply a fully connected neural network with output dimension 1 to the latent features output by the final layer:

17 $${E}_{ij}={{{{{{{{\rm{MLP}}}}}}}}}_{{{{{{{{\rm{output}}}}}}}}}({{{{{{{{\bf{x}}}}}}}}}^{ij,L={N}_{{{{{{{{\rm{layer}}}}}}}}}})$$

Finally, we note that we found normalization, both of the targets and inside the network, to be of high importance. Details are outlined in “Methods”.

### Dynamics of small molecules

We benchmark Allegro’s ability to accurately learn energies and forces of small molecules on the revised MD-17 dataset^43^, a recomputed version of the original MD-17 dataset^10,44,45^ that contains ten small, organic molecules at DFT accuracy. As shown in Table 1, Allegro obtains state-of-the-art accuracy in the mean absolute error (MAE) in force components, while NequIP performs better for the energies of some molecules. We note that while an older version of the MD-17 dataset which has widely been used to benchmark MLIPs exists^10,44,45^, it has been observed to contain noisy labels^43^ and is thus only of limited use for comparing the accuracy of MLIPs.

**Table 1:** MAE on the revised MD-17 dataset for energies and force components, in units of [meV] and [meV/Å], respectively

Molecule		FCHL19^13, 43^	UNiTE^26^	GAP^6^	ANI-pretrained^48, 49^	ANI-random^48, 49^	ACE^12^	GemNet-(T/Q)^76^	NequIP (l=3)^15^	Allegro
Aspirin	Energy	6.2	2.4	17.7	16.6	25.4	6.1	–	**2.3**	**2.3**
	Forces	20.9	7.6	44.9	40.6	75.0	17.9	9.5	8.2	**7.3**
Azobenzene	Energy	2.8	1.1	8.5	15.9	19.0	3.6	–	**0.7**	1.2
	Forces	10.8	4.2	24.5	35.4	52.1	10.9	–	2.9	**2.6**
Benzene	Energy	0.3	0.07	0.75	3.3	3.4	**0.04**	–	**0.04**	0.3
	Forces	2.6	0.73	6.0	10.0	17.5	0.5	0.5	0.3	**0.2**
Ethanol	Energy	0.9	0.62	3.5	2.5	7.7	1.2	–	**0.4**	**0.4**
	Forces	6.2	3.7	18.1	13.4	45.6	7.3	3.6	2.8	**2.1**
Malonaldehyde	Energy	1.5	1.1	4.8	4.6	9.4	1.7	–	0.8	**0.6**
	Forces	10.2	6.6	26.4	24.5	52.4	11.1	6.6	5.1	**3.6**
Naphthalene	Energy	1.2	0.46	3.8	11.3	16.0	0.9	–	**0.2**	0.5
	Forces	6.5	2.6	16.5	29.2	52.2	5.1	1.9	1.3	**0.9**
Paracetamol	Energy	2.9	1.9	8.5	11.5	18.2	4.0	–	**1.4**	1.5
	Forces	12.2	7.1	28.9	30.4	63.3	12.7	–	5.9	**4.9**
Salicylic acid	Energy	1.8	0.73	5.6	9.2	13.5	1.8	–	**0.7**	0.9
	Forces	9.5	3.8	24.7	29.7	52.0	9.3	5.3	4.0	**2.9**
Toluene	Energy	1.6	0.45	4.0	7.7	12.6	1.1	–	**0.3**	0.4
	Forces	8.8	2.5	17.8	24.3	52.9	6.5	2.2	**1.6**	1.8
Uracil	Energy	0.6	0.58	3.0	5.1	8.3	1.1	–	**0.4**	0.6
	Forces	4.2	3.8	17.6	21.4	44.1	6.6	3.8	3.1	**1.8**

Results for GAP, ANI, and ACE as reported in ref. ^24^. Best results are marked in bold. ANI-pretrained refers to a version of ANI that was pretrained on 8.9 million structures and fine-tuned on the revMD-17 dataset, ANI-random refers to a randomly initialized model trained from scratch.

### Transferability to higher temperatures

For an interatomic potential to be useful in practice, it is crucial that it be transferable to new configurations that might be visited over the course of a long molecular dynamics simulation. To assess Allegro’s generalization capabilities, we test the transferability to conformations sampled from higher-temperature MD simulations. We use the temperature transferability benchmark introduced in ref. ^24^: here, a series of data were computed using DFT for the flexible drug-like molecule 3-(benzyloxy)pyridin-2-amine (3BPA) at temperatures 300, 600, and 1200 K. Various state-of-the-art methods were trained on 500 structures from the *T* = 300 K dataset and then evaluated on data sampled at all three temperatures. Table 2 shows a comparison of Allegro against existing approaches reported in ref. ^24^: linear ACE^12^, sGDML^10^, GAP^6^, a classical force field based on the GAFF functional form^46,47^ as well as two ANI parametrizations^48,49^ (ANI-pretrained refers to a version of ANI that was pretrained on 8.9 million structures and fine-tuned on this dataset, while ANI-2x refers to the original parametrization trained on 8.9 million structures, but not fine-tuned on the 3BPA dataset). The equivariant neural networks Allegro and NequIP are observed to generalize significantly better than all other approaches.

**Table 2:** Energy and Force RMSE for the 3BPA temperature transferability dataset, reported in units of [meV] and [meV/Å]

	ACE^12^	sGDML^10^	GAP^6^	FF^46, 47^	ANI-pretrained^48, 49^	ANI-2x^48, 49^	NequIP^15^	Allegro
**Fit to 300** **K**								
300 K, E	7.1	9.1	22.8	60.8	23.5	38.6	**3.28 (0.12)**	3.84 (0.10)
300 K, F	27.1	46.2	87.3	302.8	42.8	84.4	**10.77 (0.28)**	12.98 (0.20)
600 K, E	24.0	484.8	61.4	136.8	37.8	54.5	**11.16 (0.17)**	12.07 (0.55)
600 K, F	64.3	439.2	151.9	407.9	71.7	102.8	**26.37 (0.11)**	29.11 (0.27)
1200 K, E	85.3	774.5	166.8	325.5	76.8	88.8	**38.52 (2.00)**	42.57 (1.79)
1200 K, F	187.0	711.1	305.5	670.9	129.6	139.6	**76.18 (1.36)**	82.96 (2.17)

All models were trained on *T* = 300 K. Results for all models except for NequIP and Allegro from ref. ^24^. Best results are marked in bold. For NequIP and Allegro, we report the mean over three different seeds as well as the sample standard deviation in parentheses.

### Quantum-chemical properties of small molecules

Next, we assess Allegro’s ability to accurately model properties of small molecules across chemical space using the popular QM9 dataset^50^. The QM9 dataset contains molecular properties computed with DFT of approximately 134k minimum-energy structures with chemical elements (C, H, O, N, F) that contain up to 9 heavy atoms (C, O, N, F). We benchmark Allegro on four energy-related targets, in particular: (a) *U*_0_, the internal energy of the system at *T* = 0 K, (b) *U*, the internal energy at *T* = 298.15 K, (c) *H*, the enthalpy at *T* = 298.15 K, and (d) *G*, the free energy at *T* = 298.15 K. Unlike other experiments in this work, which probed conformational degrees of freedom, we here assess the ability of Allegro to describe properties across compositional degrees of freedom. Table 3 shows a comparison with a series of state-of-the-art methods that also learn the properties described above as a direct mapping from atomic coordinates and species. We find that Allegro outperforms all existing methods. Surprisingly, even an Allegro model with a single tensor product layer obtains higher accuracy than all existing methods based on atom-centered message-passing neural networks and transformers.

**Table 3:** Comparison of models on the QM9 dataset, measured by the MAE in units of [meV]

Model	*U*_0_	*U*	*H*	*G*
Schnet^25^	14	19	14	14
DimeNet++^77^	6.3	6.3	6.5	7.6
Cormorant^23^	22	21	21	20
LieConv^78^	19	19	24	22
L1Net^79^	13.5	13.8	14.4	14.0
SphereNet^80^	6.3	7.3	6.4	8.0
EGNN^40^	11	12	12	12
ET^38^	6.2	6.3	6.5	7.6
NoisyNodes^81^	7.3	7.6	7.4	8.3
PaiNN^27^	5.9	5.7	6.0	7.4
Allegro, 1 layer	5.7 (0.3)	5.3	5.3	6.6
Allegro, 3 layers	**4.7** (0.2)	**4.4**	**4.4**	**5.7**

Allegro outperforms all existing atom-centered message-passing and transformer-based models, in particular even with a single layer. Best methods are shown in bold.

### Li-ion diffusion in a phosphate electrolyte

In order to examine Allegro’s ability to describe kinetic properties with MD simulations, we use it to study amorphous structure formation and Li-ion migration in the Li_3_PO_4_ solid electrolyte. This class of solid-state electrolytes is characterized by the intricate dependence of conductivity on the degree of crystallinity^51–54^.

**Fig. 2:**
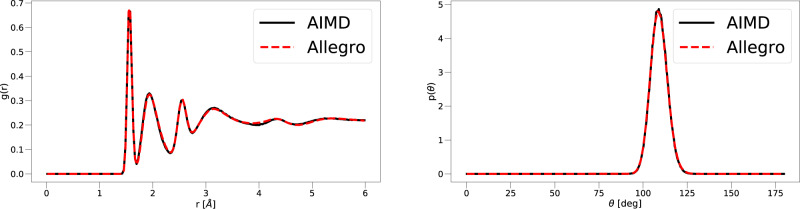
Structural properties of Li_3_PO_4_. Left: radial distribution function, right: angular distribution function of tetrahedral bond angle. All defined as probability density functions. Results from Allegro are shown in red, and those from AIMD are shown in black.

**Fig. 3:**
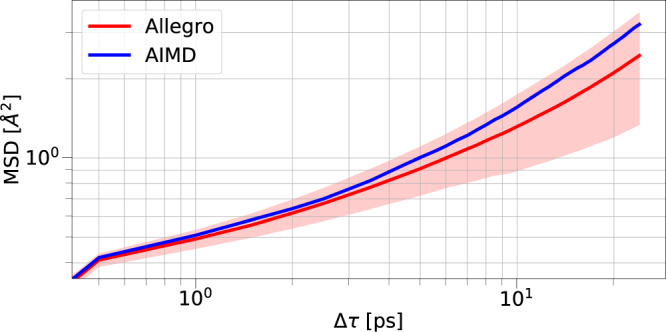
Li dynamics in Li_3_PO_4_. Comparison of the Li MSD of AIMD vs. Allegro. Results are averaged over 10 runs of Allegro, shading indicates +/– one standard deviation. Results from Allegro are shown in red, and those from AIMD are shown in blue.

**Fig. 4:**
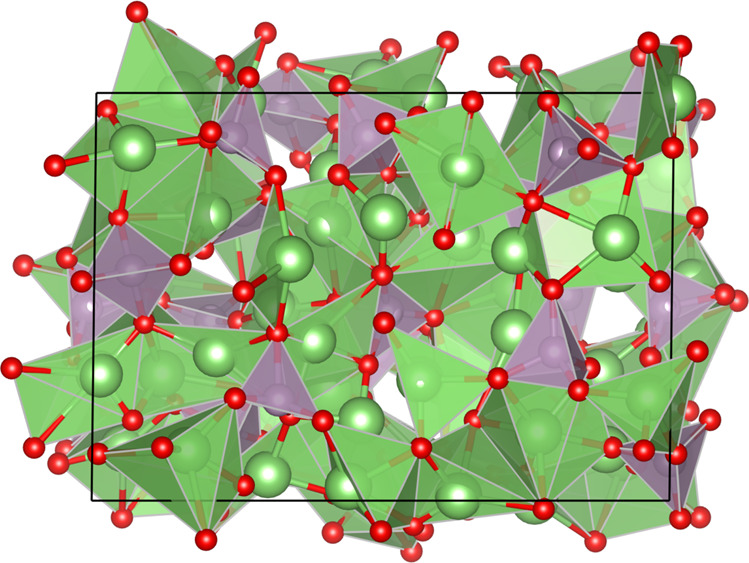
Structure of Li_3_PO_4_. The quenched Li_3_PO_4_ structure at *T* = 600 K.

In particular, the Li_3_PO_4_ dataset used in this work consists of two parts: a 50 ps ab-initio molecular dynamics (AIMD) simulation in the molten liquid state at *T* = 3000 K, followed by a 50 ps AIMD simulation in the quenched state at *T* = 600 K. We train a potential on structures from the liquid and quenched trajectories. The model used here is computationally efficient due to a relatively small number of parameters (9058 weights) and tensor products. In particular, we note that the model used to measure the faithfulness of the kinetics and to measure Allegro’s ability to predict thermodynamic observables is identical to the one used in scaling experiments detailed below. This is crucial for fair assessment of a method that simultaneously scales well and can accurately predict material properties. When evaluated on the test set for the quenched amorphous state, which the simulation is performed on, a MAE in the energies of 1.7 meV/atom was obtained, as well as a MAE in the force components of 73.4 meV/Å. We then run a series of ten MD simulations starting from the initial structure of the quenched AIMD simulation, all of length 50 ps at *T* = 600 K in the quenched state, in order to examine how well Allegro recovers the structure and kinetics compared to AIMD. To assess the quality of the structure after the phase change, we compare the all-atom radial distribution functions (RDF) and the angular distribution functions (ADF) of the tetrahedral angle P–O–O (P central atom). We show in Fig. 2 that Allegro can accurately recover both distribution functions. For the aspect of ion transport kinetics, we test how well Allegro can model the Li mean-square-displacement (MSD) in the quenched state. We again find excellent agreement with AIMD, as shown in Fig. 3. The structure of Li_3_PO_4_ can be seen Fig. 4.

### Scaling

Many interesting phenomena in materials science, chemistry, and biology require large numbers of atoms, long timescales, a diversity of chemical elements, or often all three. Scaling to large numbers of atoms requires parallelization across multiple workers, which is difficult in atom-centered MPNNs because the iterative propagation of atomic state information along the atomistic graph increases the size of the receptive field as a function of the number of layers. This is further complicated by the fact that access to energy-conservative force fields requires computing the negative gradient of the predicted energy, which in standard backpropagation algorithms also requires propagating gradient information along the atom graph. Allegro is designed to avoid this issue by strict locality. A given Allegro model scales as:

- ${{{{{{{\mathcal{O}}}}}}}}(N)$ in the number of atoms in the system *N*, in contrast to the ${{{{{{{\mathcal{O}}}}}}}}({N}^{2})$ scaling of some global descriptor methods such as sGDML^10^;
- ${{{{{{{\mathcal{O}}}}}}}}(M)$ in the number of neighbors per atom *M*, in contrast to the quadratic ${{{{{{{\mathcal{O}}}}}}}}({M}^{2})$ scaling of some deep-learning approaches such as DimeNet^14^ or Equivariant Transformers^38,55^;
- ${{{{{{{\mathcal{O}}}}}}}}(1)$ in the number of species *S*, unlike local descriptors such as SOAP (${{{{{{{\mathcal{O}}}}}}}}({S}^{2})$) or ACE (${{{{{{{\mathcal{O}}}}}}}}({S}^{{{{{{{{\rm{bodyorder}}}}}}}}-1})$)^12^.

We note, however, that the per-pair featurization of Allegro has larger memory requirements than if one were to choose the same number of features in a per-atom featurization. In practice, we find this to not be a problem and see that Allegro can be scaled to massive systems by parallelizing over modest computational resources.

In particular, in addition to scaling as ${{{{{{{\mathcal{O}}}}}}}}(N)$ in the number of atoms, Allegro is strictly local within the chosen cutoff and thus easy to parallelize in large-scale calculations. Recall that Eqs. (5) and (6) define the total energy of a system in Allegro as a sum over scaled pairwise energies *E*_*ij*_. Thus by linearity, the force on atom *a*

$${\overrightarrow{F}}_{a}=-{\nabla }_{a}{E}_{{{{{{{{\rm{system}}}}}}}}}=-\mathop{\sum}\limits_{i,j}{\nabla }_{a}{E}_{ij},$$

ignoring the per-species and per-species-pair scaling coefficients ${\sigma }_{{Z}_{i}}$ and ${\sigma }_{{Z}_{i},{Z}_{j}}$ for clarity. Because each *E*_*ij*_ depends only the atoms in the neighborhood of atom *i*, − ∇_*a*_*E*_*ij*_ ≠ 0 only when *a* is in the neighborhood of *i*. Further, for the same reason, pair energy terms *E*_*ij*_ with different central atom indices *i* are independent. As a result, these groups of terms may be computed independently for each central atom, which facilitates parallelization: the contributions to the force on atom *a* due to the neighborhoods of various different atoms can be computed in parallel by whichever worker is currently assigned the relevant center’s neighborhood. The final forces are then simple sum reductions over force terms from various parallel workers.

We first demonstrate the favorable scaling of Allegro in system size by parallelizing the method across GPUs on a single compute node as well as across multiple GPU nodes. We choose two test systems for the scaling experiments: (a) the quenched state structures of the multi-component electrolyte Li_3_PO_4_ and (b) the Ag bulk crystal with a vacancy, simulated at 90% of the melting temperature. The Ag model used 1000 structures for training and validation, resulting in energy MAE of 0.397 meV/atom and force MAE of 16.8 meV/Å on a test set of 159 structures. Scaling numbers are dependent on a variety of hyperparameter choices, such as network size and radial cutoff, that control the trade-off between evaluation speed and accuracy. For Li_3_PO_4_, we explicitly choose these identically to those used in the previous set of experiments in order to demonstrate how well an Allegro potential scales that we demonstrated to give highly accurate prediction of structure and kinetics. Table 4 shows the computational efficiency for varied size and computational resources. We are able to simulate the Ag system with over 100 million atoms on 16 GPU nodes.

**Table 4:** Simulation times obtainable in [ns/day] and time required per atom per step in [microseconds] for varying number of atoms and computational resources

Material	Number of atoms	Number of GPUs	Speed in ns/day	Microseconds/(atom ⋅ step)
Li_3_PO_4_	192	1	32.391	27.785
Li_3_PO_4_	421,824	1	0.518	0.552
Li_3_PO_4_	421,824	2	1.006	0.284
Li_3_PO_4_	421,824	4	1.994	0.143
Li_3_PO_4_	421,824	8	3.810	0.075
Li_3_PO_4_	421,824	16	6.974	0.041
Li_3_PO_4_	421,824	32	11.530	0.025
Li_3_PO_4_	421,824	64	15.515	0.018
Li_3_PO_4_	50,331,648	128	0.274	0.013
Ag	71	1	90.190	67.463
Ag	1,022,400	1	1.461	0.289
Ag	1,022,400	2	2.648	0.160
Ag	1,022,400	4	5.319	0.079
Ag	1,022,400	8	10.180	0.042
Ag	1,022,400	16	18.812	0.022
Ag	1,022,400	32	28.156	0.015
Ag	1,022,400	64	43.438	0.010
Ag	1,022,400	128	49.395	0.009
Ag	100,640,512	128	1.539	0.003

Time steps of 2fs and 5fs were used for Li_3_PO_4_ and Ag, respectively.

**Fig. 5:**
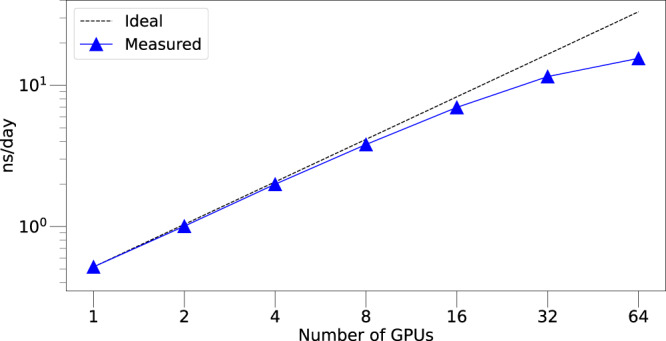
Scaling results. Strong scaling results on a Li_3_PO_4_ structure of 421,824 atoms, performed in LAMMPS.

The parallel nature of the method and its implementation also allows multiple GPUs to be used to increase the speed of the potential calculation for a fixed-size system. Figure 5 shows such strong scaling results on a 421,824 atom Li_3_PO_4_ structure. The system size was kept constant while varying the number of A100 GPUs.

### Theoretical analysis

In this section, we provide a theoretical analysis of the method by highlighting similarities and differences to the Atomic Cluster Expansion (ACE) framework^12^. Throughout this section we omit representation indices *ℓ* and *p* from the notation for conciseness: every weight or feature that carries *ℓ* and *p* indices previously implicitly carries them in this section. Starting from the initial equivariant features for the pair of atoms *ij* at layer *L* = 0

18 $${{{{{{{{\bf{V}}}}}}}}}_{{n}_{0}}^{ij,L=0}={w}_{{n}_{0}}^{ij,L=0}{\overrightarrow{Y}}^{ij}$$

the first Allegro layer computes a sum over tensor products between ${{{{{{{{\bf{V}}}}}}}}}_{{n}_{0}}^{ij,L=0}$ and the spherical harmonics projection of all neighbors $k\in {{{{{{{\mathcal{N}}}}}}}}(i)$:

19 $${{{{{{{{\bf{V}}}}}}}}}_{{n}_{1}}^{ij,L=1}=\mathop{\sum}\limits_{\begin{array}{c}{n}_{1}^{{\prime} }\\ {{{{{{{\rm{paths}}}}}}}}\end{array}}{w}_{{n}_{1},{n}_{1}^{{\prime} },{{{{{{{\rm{path}}}}}}}}}^{L=1}\mathop{\sum}\limits_{{k}_{1}\in {{{{{{{\mathcal{N}}}}}}}}(i)}{w}_{{n}_{1}^{{\prime} }}^{i{k}_{1},L=1}\left({w}_{{n}_{1}^{{\prime} }}^{ij,L=0}{\overrightarrow{Y}}^{ij}\otimes {\overrightarrow{Y}}^{i{k}_{1}}\right)$$

20 $$=\mathop{\sum}\limits_{\begin{array}{c}{n}_{1}^{{\prime} }\\ {{{{{{{\rm{paths}}}}}}}}\end{array}}{w}_{{n}_{1},{n}_{1}^{{\prime} },{{{{{{{\rm{path}}}}}}}}}^{L=1}\mathop{\sum}\limits_{{k}_{1}\in {{{{{{{\mathcal{N}}}}}}}}(i)}{w}_{{n}_{1}^{{\prime} }}^{i{k}_{1},L=1}{w}_{{n}_{1}^{{\prime} }}^{ij,L=0}\Big({\overrightarrow{Y}}^{ij}\otimes {\overrightarrow{Y}}^{i{k}_{1}}\Big)$$

which follows from the bilinearity of the tensor product. The sum over “paths” in this equation indicates the sum over all symmetrically valid combinations of implicit irrep indices on the various tensors present in the equation as written out explicitly in Eq. (16). Repeating this substitution, we can express the equivariant features at layer *L* = 2 and reveal a general recursive relationship:

21 $${{{{{{{{\bf{V}}}}}}}}}_{{n}_{2},{\ell }_{2},{p}_{2}}^{ij,L=2}=\mathop{\sum}\limits_{\begin{array}{c}{n}_{2}^{{\prime} }\\ {{{{{{{\rm{paths}}}}}}}}\end{array}}{w}_{{n}_{2},{n}_{2}^{{\prime} },{{{{{{{\rm{path}}}}}}}}}^{L=2}\mathop{\sum}\limits_{{k}_{2}\in {{{{{{{\mathcal{N}}}}}}}}(i)}{w}_{{n}_{2}^{{\prime} }}^{i{k}_{2},L=2}\left({{{{{{{{\bf{V}}}}}}}}}_{{n}_{2}^{{\prime} }}^{ij,L=1}\otimes {\overrightarrow{Y}}^{i{k}_{2}}\right)$$

22 $$=\mathop{\sum}\limits_{\begin{array}{c}{n}_{1}^{{\prime} },{n}_{2}^{{\prime} }\\ {{{{{{{\rm{paths}}}}}}}}\end{array}}{w}_{{n}_{2},{n}_{2}^{{\prime} },{{{{{{{\rm{path}}}}}}}}}^{L=2}{w}_{{n}_{2}^{{\prime} },{n}_{1}^{{\prime} },{{{{{{{\rm{path}}}}}}}}}^{L=1}\left[\mathop{\sum}\limits_{{k}_{2}\in {{{{{{{\mathcal{N}}}}}}}}(i)}\mathop{\sum}\limits_{{k}_{1}\in {{{{{{{\mathcal{N}}}}}}}}(i)}{w}_{{n}_{2}^{{\prime} }}^{i{k}_{2},L=2}{w}_{{n}_{1}^{{\prime} }}^{i{k}_{1},L=1}{w}_{{n}_{1}^{{\prime} }}^{ij,L=0}\left({\overrightarrow{Y}}^{ij}\otimes {\overrightarrow{Y}}^{i{k}_{1}}\otimes {\overrightarrow{Y}}^{i{k}_{2}}\right)\right]$$

23 $${{{{{{{{\bf{V}}}}}}}}}_{{n}_{L},{\ell }_{L},{p}_{L}}^{ij,L}=\mathop{\sum}\limits_{\begin{array}{c}{k}_{1},\ldots,{k}_{L}\\ {n}_{1}^{{\prime} },\ldots,{n}_{L}^{{\prime} }\\ {{{{{{{\rm{paths}}}}}}}}\end{array}}\left[\left(\mathop{\prod}\limits_{\alpha \in 1,\ldots,L}{w}_{{n}_{\alpha+1}^{{\prime} },{n}_{\alpha }^{{\prime} },{{{{{{{\rm{path}}}}}}}}}^{L=\alpha }\right)\left(\mathop{\prod}\limits_{\alpha \in 0,\ldots,L}{w}_{{n}_{\alpha }^{{\prime} }}^{i{k}_{\alpha },L=\alpha }\right)\left(\mathop{\bigotimes}\limits_{\alpha \in 0,\ldots,L}{\overrightarrow{Y}}^{i{k}_{\alpha }}\right)\right]$$

where *k*_0_ = *j*, $${n}_{L+1}^{{\prime} }={n}_{L}$$, and $${n}_{0}^{{\prime} }={n}_{1}^{{\prime} }$$.

The ACE descriptor ${B}_{{n}_{1}...{n}_{\nu }}^{(\nu )}$ of body order *ν* + 1^12^ can also be written as an iterated tensor product, specifically of the projection *A*_*n*_ of the local atomic density onto a spherical harmonic and radial–chemical basis. The *n* index here runs over the *N*_full-basis_ = *S* × *N*_basis_ combined radial–chemical basis functions. Starting from this definition we may again use the bilinearity of the tensor product to expand the ACE descriptor:

24 $${B}_{{n}_{1}...{n}_{\nu }}^{(\nu )}=\mathop{\bigotimes}\limits_{\alpha=1,...,\nu }{A}_{{n}_{i}}$$

25 $$=\mathop{\bigotimes}\limits_{\alpha=1,...,\nu }\left(\mathop{\sum}\limits_{{k}_{\alpha }\in {{{{{{{\mathcal{N}}}}}}}}(i)}{R}_{{n}_{\alpha }}({r}_{i{k}_{\alpha }},{z}_{{k}_{\alpha }}){\overrightarrow{Y}}^{i{k}_{\alpha }}\right)$$

26 $$=\mathop{\sum}\limits_{{k}_{1},...,{k}_{\nu }}\left[\left(\mathop{\prod}\limits_{\alpha \in 1,...,\nu }{R}_{{n}_{\alpha }}({r}_{i{k}_{\alpha }},{z}_{{k}_{\alpha }})\right)\left(\mathop{\bigotimes}\limits_{\alpha \in 1,...,\nu }{\overrightarrow{Y}}^{i{k}_{\alpha }}\right)\right]$$

Comparing Eqs. (23) and (26) it is immediately evident that an Allegro model with *N*_layer_ layers and an ACE expansion of body order *ν* + 1 = *N*_layer_ + 2 share the core equivariant iterated tensor products ${\overrightarrow{Y}}^{ij}\otimes {\overrightarrow{Y}}^{i{k}_{1}}\otimes ...\otimes {\overrightarrow{Y}}^{i{k}_{{N}_{{{{{{{{\rm{layer}}}}}}}}}}}$. The equivariant Allegro features ${{{{{{{{\bf{V}}}}}}}}}_{n}^{ij,L}$ are analogous—but not equivalent—to the full equivariant ACE basis functions ${B}_{{n}_{1}...{n}_{L+1}}^{(L+1)}$.

The comparison of these expansions of the two models emphasizes, as discussed earlier in the scaling section, that the ACE basis functions carry a full set of *n*_*α*_ indices (which label radial–chemical two-body basis functions), the number of which increases at each iteration, while the Allegro features do not exhibit this increase as a function of the number of layers. This difference is the root of the contrast between the ${{{{{{{\mathcal{O}}}}}}}}({N}_{{{{{{{{\rm{full-basis}}}}}}}}}^{\nu })$ scaling of ACE in the size of the radial–chemical basis *N*_full-basis_ and the ${{{{{{{\mathcal{O}}}}}}}}(1)$ of Allegro. Allegro achieves this more favorable scaling through the learnable channel mixing weights.

A key difference between Allegro and ACE, made clear here, is their differing construction of the scalar pairwise weights. In ACE, the scalar weights carrying *ik*_*α*_ indices are the radial–chemical basis functions *R*, which are two-body functions of the distance between atoms *i* and *k*_*α*_ and their chemistry. These correspond in Allegro to the environment embedding weights ${w}_{i{k}_{\alpha },n}^{L}$, which—critically—are functions of all the lower-order equivariant features ${{{{{{{{\bf{V}}}}}}}}}_{n}^{ij,{L}^{{\prime} } < L}$: the environment embedding weights at layer *L* are a function of the scalar features from layer *L* − 1 (Eq. (14)) which are a function of the equivariant features from layer *L* − 2 (Eq. (15)) and so on. As a result, the “pairwise” weights have a hierarchical structure and depend on all previous weights:

27 $${w}_{ix,n}^{L}=f({{{{{{{{\bf{V}}}}}}}}}^{ix,L-1})$$

28 $$=f\left(\{{w}_{i{x}^{{\prime} },{n}^{{\prime} }}^{{L}^{{\prime} }}\,{{{{{{{\rm{for\,all}}}}}}}}\,{n}^{{\prime} },{L}^{{\prime} } \, < \,L,{x}^{{\prime} }\in {{{{{{{\mathcal{N}}}}}}}}(i)\}\right)$$

where *f* contains details irrelevant to conveying the existence of the dependence. We hypothesize that this hierarchical nature is in part of why Allegro performs so much better than the ACE model and is a key difference to ACE and its variants, such as NICE. We finally note that the expanded features of Eq. (23)—and thus the final features of any Allegro model—are of finite body order if the environment embedding weights ${w}_{i{k}_{\alpha }n}^{L}$ are themselves of finite body order. This condition holds if the latent and embedding MLPs are linear. If any of these MLPs contain nonlinearities whose Taylor expansions are infinite, the body order of the environment embedding weights, and thus of the entire model, becomes infinite. Nonlinearities in the two-body MLP are not relevant to the body order and correspond to the use of a nonlinear radial basis in methods such as ACE. Allegro models whose only nonlinearities lie in the two-body embedding MLP are still highly accurate and such a model was used in the experiments on the 3BPA dataset described above.

## Discussion

A new type of deep-learning interatomic potential is introduced that combines high prediction accuracy on energies and forces, enabled by its equivariant architecture, with the ability to scale to large system sizes, due to the strict locality of its geometric representations. The Allegro method surpasses the state-of-the-art set by atom-centered message-passing neural network models for interatomic interactions in terms of combined accuracy and scalability. This makes it possible to predict structural and kinetic properties from molecular dynamics simulations of complex systems of millions of atoms at nearly first-principles fidelity.

Our findings enable the study of molecular and materials system with equivariant neural networks that were previously inaccessible and raise broad questions about the optimal choice of representation and learning algorithm for machine learning on molecules and materials. We note that the Allegro method naturally offers a trade-off between accuracy and computational speed, while still offering efficient parallel scalability. Models of higher accuracy can be obtained by choosing networks with higher capacity (including larger numbers of features and more layers), but we also found a small, fast model to work sufficiently well to capture complex structural and kinetic properties in our example applications. It would be of great value to the community to conduct a detailed analysis of this accuracy-speed trade-off across different machine learning interatomic potentials and materials.

The correspondences between the Allegro architecture and the atomic cluster expansion (ACE) formalism also raise questions about how and why Allegro is able to outperform the systematic ACE basis expansion. We speculate that our method’s performance is due in part to the learned dependence of the environment embedding weights at each layer on the full scalar latent features from all previous layers. This dependence may allow the importance of an atom to higher body-order interactions to be learned as a function of lower body-order descriptions of its environment. It stands in stark contrast to ACE, where the importance of any higher body-order interaction is learned separately from lower body-order descriptions of the local structure. We believe further efforts to understand this correspondence are a promising direction for future work. Similarly, we believe a systematic study of the completeness of the prescribed architecture will be of high interest.

Another important goal for future work is to obtain a better understanding of when explicit long-range terms are required in machine learning interatomic potentials, how to optimally incorporate them with local models, and to what extent message-passing interatomic potentials may or may not implicitly capture these interactions. For example, it would be interesting to combine the Allegro potential with an explicit long-range energy term. In particular, the strict locality of the Allegro model naturally facilitates separation of the energy into a short-range term and a physically motivated long-range term.

## Methods

### Software

All experiments were run with the Allegro code available at https://github.com/mir-group/allegro under git commit a5128c2a86350762215dad6bd8bb42875ebb06cb. In addition, we used the NequIP code available at https://github.com/mir-group/nequip with version 0.5.3, git commit eb6f9bca7b36162abf69ebb017049599b4ddb09c, as well as e3nn with version 0.4.4^56^, PyTorch with version 1.10.0^57^, and Python with version 3.9.7. The LAMMPS experiments were run with the LAMMPS code available at https://github.com/lammps/lammps.git under git commit 9b989b186026c6fe9da354c79cc9b4e152ab03af with the pair_allegro code available at https://github.com/mir-group/pair_allegro, git commit 0161a8a8e2fe0849165de9eeae3fbb987b294079. The VESTA software was used to generate Fig. 4^58^. Matplotlib was used for plotting results^59^.

### Reference training sets

#### revised MD-17

The revised MD-17 dataset consists of ten small organic molecules, for which 100,000 structures were computed at DFT (PBE/def2-SVP) accuracy using a very tight SCF convergence and very dense DFT integration grid^43^. The structures were recomputed from the original MD-17 dataset^10,44,45^. The data can be obtained at https://figshare.com/articles/dataset/Revised_MD17_dataset_rMD17_/12672038. We use 950 structures for training, 50 structures for validation (both sampled randomly), and evaluate the test error on all remaining structures.

#### 3BPA

The 3BPA dataset consists of 500 training structures at *T* = 300 K, and test data at 300 K, 600 K, and 1200 K, of dataset size of 1669, 2138, and 2139 structures, respectively. The data were computed using Density Functioal Theory with the *ω*B97X exchange-correlation functional and the 6-31G(d) basis set. For details, we refer the reader to^24^. The dataset was downloaded from https://pubs.acs.org/doi/full/10.1021/acs.jctc.1c00647.

#### QM9

The QM9 data consist of 133,885 structures with up to 9 heavy elements and consisting of species H, C, N, O, F in relaxed geometries. Structures are provided together with a series of properties computed at the DFT/B3LYP/6-31G(2df,p) level of theory. The dataset was downloaded from https://figshare.com/collections/Quantum_chemistry_structures_and_properties_of_134_kilo_molecules/978904. In line with previous work, we excluded the 3054 structures that failed the geometry consistency check, resulting in 130,831 total structures, of which we use 110,000 for training, 10,000 for validation and evaluate the test error on all remaining structures. Training was performed in units of [eV].

#### Li_3_PO_4_

The Li_3_PO_4_ structure consists of 192 atoms. The reference dataset was obtained from two AIMD simulations both of 50 ps duration, performed in the Vienna Ab-Initio Simulation Package (VASP)^60–62^ using a generalized gradient PBE functional^63^, projector augmented wave pseudopotentials^64^, a plane-wave cutoff of 400 eV and a Γ-point reciprocal-space mesh. The integration was performed with a time step of 2 fs in the NVT ensemble using a Nosé–Hoover thermostat. The first 50 ps of the simulation were performed at *T* = 3000 K in the molten phase, followed by an instant quench to *T* = 600 K and a second 50 ps simulation at *T* = 600 K. The two trajectories were combined and the training set of 10,000 structures as well as the validation set of 1000 were sampled randomly from the combined dataset of 50,000 structures.

#### Ag

The Ag system is created from a bulk face-centered-cubic structure with a vacancy, consisting of 71 atoms. The data were sampled from AIMD simulations at *T* = 1111 K (90% of the melting temperature of Ag) with Gamma-point k-sampling as computed in VASP using the PBE exchange-correlation functional^60–62^. Frames were then extracted at least 25 fs apart, to limit correlation within the trajectory, and each frame was recalculated with converged DFT parameters. For these calculations, the Brillouin zone was sampled using a (2 × 2 × 3) Gamma-centered k-point grid, and the electron density at the Fermi-level was approximated using Methfessel–Paxton smearing^65^ with a sigma value of 0.05. A cutoff energy of 520 eV was employed, and each calculation was non-spin-polarized.

### Molecular dynamics simulations

Molecular Dynamics simulations were performed in LAMMPS^66^ using the pair style pair_allegro implemented in the Allegro interface, available at https://github.com/mir-group/pair_allegro. We run the Li_3_PO_4_ production and timing simulations under an NVT ensemble at *T* = 600 K, using a time step of 2 fs, a Nosé-Hoover thermostat and a temperature damping parameter of 40 time steps. The Ag timing simulations are run also in NVT, at a temperature of *T* = 300 K using a time step of 5 fs, a Nosé-Hoover thermostat and a temperature damping parameter of 40 time steps. The larger systems are created by replicating the original structures of 192 and 71 atoms of Li_3_PO_4_ and Ag, respectively. We compute the RDF and ADFs for Li_3_PO_4_ with a maximum distance of 10 Å (RDF) and 2.5 Å (ADFs). We start the simulation from the first frame of the AIMD quench simulation. RDF and ADF for Allegro were averaged over ten runs with different initial velocities, the first 10 ps of the 50 ps simulation were discarded in the RDF/ADF analysis to account for equilibration.

### Training details

Models were trained on a NVIDIA V100 GPU in single-GPU training.

#### revMD-17 and 3BPA

The revised MD-17 models were trained with a total budget of 1000 structures, split into 950 for training and 50 for validation. The 3BPA model was trained with a total budget of 500 structures, split into 450 for training and 50 for validation. The dataset was re-shuffled after each epoch. We use three layers, 128 features for both even and odd irreps and a *ℓ*_max_ = 3. The 2-body latent MLP consists of four hidden layers of dimensions [128, 256, 512, 1024], using SiLU nonlinearities on the outputs of the hidden layers^67^. The later latent MLPs consist of three hidden layers of dimensionality [1024, 1024, 1024] using SiLU nonlinearities for revMD-17 and no nonlinearities for 3BPA. The embedding weight projection was implemented as a single matrix multiplication without a hidden layer or a nonlinearity. The final edge energy MLP has one hidden layer of dimension 128 and again no nonlinearity. All four MLPs were initialized according to a uniform distribution of unit variance. We used a radial cutoff of 7.0 Å for all molecules in the revMD-17 dataset, except for naphthalene, for which a cutoff of 9.0 Å was used, and a cutoff of 5.0 Å for the 3BPA dataset. We have also included an ablation study on the cutoff radius for the large naphthalene molecule which can be found in Supplementary Table 1. We use a basis of eight non-trainable Bessel functions for the basis encoding with the polynomial envelope function using *p* = 6 for revMD-17 and *p* = 2 for 3BPA. We found it particularly important to use a low exponent *p* in the polynomial envelope function for the 3BPA experiments. We hypothesize that this is due to the fact that a lower exponent provides a stronger decay with increasing interatomic distance (see Supplementary Fig. 1), thereby inducing a stronger inductive bias that atoms *j* further away from a central atom *i* should have smaller pair energies *E*_*ij*_ and thus contribute less to atom *i*’s site energy *E*_*i*_. RevMD-17 models were trained using a joint loss function of energies and forces:

29 $${{{{{{{\mathcal{L}}}}}}}}=\frac{{\lambda }_{E}}{B}\mathop{\sum }\limits_{b}^{B}{\left({\hat{E}}_{b}-{E}_{b}\right)}^{2}+\frac{{\lambda }_{F}}{3BN}\mathop{\sum }\limits_{i=1}^{BN}\mathop{\sum }\limits_{\alpha=1}^{3}\left|\left|-\frac{\partial \hat{E}}{\partial {r}_{i,\alpha }}-{F}_{i,\alpha }\right|\right|^{2}$$

where *B*, *N*, *E*_*b*_, ${\hat{E}}_{b}$, *F*_*i*,*α*_ denote the batch size, number of atoms, batch of true energies, batch of predicted energies, and the force component on atom *i* in spatial direction *α*, respectively and *λ*_*E*_, *λ*_*F*_ are energy and force weights. Following previous works, for the revMD-17 data the force weight was set to 1000 and the weight on the total potential energies was set to 1. For the 3BPA molecules, as in ref. ^68^, we used a per-atom MSE term that divides the energy term by ${N}_{atoms}^{2}$ because (a) the potential energy is a global size-extensive property, and (b) we use a MSE loss function:

30 $${{{{{{{\mathcal{L}}}}}}}}=\frac{{\lambda }_{E}}{B}\mathop{\sum }\limits_{b}^{B}{\left(\frac{{\hat{E}}_{b}-{E}_{b}}{N}\right)}^{2}+\frac{{\lambda }_{F}}{3BN}\mathop{\sum }\limits_{i=1}^{BN}\mathop{\sum }\limits_{\alpha=1}^{3}\left|\left|-\frac{\partial \hat{E}}{\partial {r}_{i,\alpha }}-{F}_{i,\alpha }\right|\right|^{2}$$

After this normalization, both the energy and the force term receive a weight of 1. Models were trained with the Adam optimizer^69^ in PyTorch^57^, with default parameters of *β*_1_ = 0.9, *β*_2_ = 0.999, and *ϵ* = 10^−8^ without weight decay. We used a learning rate of 0.002 and a batch size of 5. The learning rate was reduced using an on-plateau scheduler based on the validation loss with a patience of 100 and a decay factor of 0.8. We use an exponential moving average with weight 0.99 to evaluate on the validation set as well as for the final model. Training was stopped when one of the following conditions was reached: (a) a maximum training time of 7 days, (b) a maximum number of epochs of 100,000, (c) no improvement in the validation loss for 1000 epochs, (d) the learning rate dropped lower than 1e-6. We note that such long wall times are usually not required and highly accurate models can typically be obtained within a matter of hours or even minutes. All models were trained with float32 precision.

#### 3BPA, NequIP

The NequIP models on the 3BPA dataset were trained with a total budget of 500 molecules, split into 450 for training and 50 for validation. The dataset was re-shuffled after each epoch. We use 5 layers, 64 features for both even and odd irreps and a *ℓ*_max_ = 3. We use a radial network of three layers with 64 hidden neurons and SiLU nonlinearities. We further use equivariant, SiLU-based gate nonlinearities as outlined in ref. ^15^, where even and odd scalars are not gated, but operated on directly by SiLU and tanh nonlinearities, respectively. We used a radial cutoff of 5.0 Å and a non-trainable Bessel basis of size 8 for the basis encoding with a polynomial envelope function using *p* = 2. Again, a low *p* value was found to be important. We use again a per-atom MSE loss function in which both the energy and the force term receive a weight of 1. Models were trained with Adam with the AMSGrad variant in the PyTorch implementation^57,69–71^, with default parameters of *β*_1_ = 0.9, *β*_2_ = 0.999, and *ϵ* = 10^−8^ without weight decay. We used a learning rate of 0.01 and a batch size of 5. The learning rate was reduced using an on-plateau scheduler based on the validation loss with a patience of 50 and a decay factor of 0.8. We use an exponential moving average with weight 0.99 to evaluate on the validation set as well as for the final model. Training was stopped when one of the following conditions was reached: (a) a maximum training time of 7 days, (b) a maximum number of epochs of 100,000, (c) no improvement in the validation loss for 1000 epochs, (d) the learning rate dropped lower than 1e-6. We note that such long wall times are usually not required and highly accurate models can typically be obtained within a matter of hours or even minutes. All models were trained with float32 precision. We use a per-atom shift ${\mu }_{{Z}_{i}}$ via the average per-atom potential energy over all training frames and a per-atom scale ${\sigma }_{{Z}_{i}}$ as the root-mean-square of the components of the forces over the training set.

#### Li_3_PO_4_

The Li_3_PO_4_ model was trained with a total budget of 11,000 structures, split into 10,000 for training and 1000 for validation. The dataset was re-shuffled after each epoch. We use one layer, 1 feature of even parity and *ℓ*_max_ = 1. The 2-body latent MLP consists of 2 hidden layers of dimensions [32, 64], using SiLU nonlinearities^67^. The later latent MLP consist of 1 hidden layer of dimensionality [64], also using a SiLU nonlinearity. The embedding weight projection was implemented as a single matrix multiplication without a hidden layer or a nonlinearity. The final edge energy MLP has one hidden layer of dimension 32 and again no nonlinearity. All four MLPs were initialized according to a uniform distribution of unit variance. We used a radial cutoff of 4.0 Å and a basis of eight non-trainable Bessel functions for the basis encoding with the polynomial envelope function using *p* = 48. The model was trained using a joint loss function of energies and forces. We use again the per-atom MSE as describe above and a weighting of 1 for the force term and 1 for the per-atom MSE term. The model was trained with the Adam optimizer^69^ in PyTorch^57^, with default parameters of *β*_1_ = 0.9, *β*_2_ = 0.999, and *ϵ* = 10^−8^ without weight decay. We used a learning rate of 0.001 and a batch size of 1. The learning rate was reduced using an on-plateau scheduler based on the validation loss with a patience of 25 and a decay factor of 0.5. We use an exponential moving average with weight 0.99 to evaluate on the validation set as well as for the final model. Training was stopped when one of the following conditions was reached: (a) a maximum training time of 7 days, (b) a maximum number of epochs of 100,000, (c) no improvement in the validation loss for 1000 epochs, (d) the learning rate dropped lower than 1e-5. The model was trained with float32 precision.

#### Ag

The Ag model was trained with a total budget of 1000 structures, split into 950 for training and 50 for validation, and evaluated on a separate test set of 159 structures. The dataset was re-shuffled after each epoch. We use 1 layer, 1 feature of even parity and *ℓ*_max_ = 1. The 2-body latent MLP consists of 2 hidden layers of dimensions [16, 32], using SiLU nonlinearities^67^. The later latent MLP consists of 1 hidden layer of dimensionality [32], also using a SiLU nonlinearity. The embedding weight projection was implemented as a single matrix multiplication without a hidden layer or a nonlinearity. The final edge energy MLP has one hidden layer of dimension 32 and again no nonlinearity. All four MLPs were initialized according to a uniform distribution. We used a radial cutoff of 4.0 Å and a basis of eight non-trainable Bessel functions for the basis encoding with the polynomial envelope function using *p* = 48. The model was trained using a joint loss function of energies and forces. We use again the per-atom MSE as describe above and a weighting of 1 for the force term and 1 for the per-atom MSE term. The model was trained with the Adam optimizer^69^ in PyTorch^57^, with default parameters of *β*_1_ = 0.9, *β*_2_ = 0.999, and *ϵ* = 10^−8^ without weight decay. We used a learning rate of 0.001 and a batch size of 1. The learning rate was reduced using an on-plateau scheduler based on the validation loss with patience of 25 and a decay factor of 0.5. We use an exponential moving average with weight 0.99 to evaluate on the validation set as well as for the final model. The model was trained for a total of approximately 5 h with float32 precision.

#### QM9

We used 110,000 molecular structures for training, 10,000 for validation, and evaluated the test error on all remaining structures, in line with previous approaches^9,27^. We note that Cormorant and EGNN are trained on 100,000 structures, L1Net is trained on 109,000 structures while NoisyNodes is trained on 114,000 structures. To give an estimate of the variability of training as a function of random seed, we report for the *U*_0_ target the mean and sample standard deviation across three different random seeds, resulting in different samples of training set as well as different weight initialization. We report two models, one with three layers and *ℓ*_max_ = 2 and another one with 1 layer and *ℓ*_max_ = 3, both with 256 features for both even and odd irreps. The 1-layer and 3-layer networks have 7,375,237 and 17,926,533 parameters, respectively. The 2-body latent MLP consists of four hidden layers of dimensions [128, 256, 512, 1024], using SiLU nonlinearities^67^. The later latent MLPs consist of three hidden layers of dimensionality [1024, 1024, 1024], also using SiLU nonlinearities. The embedding weight projection was implemented as a single matrix multiplication without a hidden layer or a nonlinearity. The final edge energy MLP has one hidden layer of dimension 128 and again no nonlinearity. All four MLPs were initialized according to a uniform distribution. We used a radial cutoff of 10.0 Å. We use a basis of 8 non-trainable Bessel functions for the basic encoding with the polynomial envelope function using *p* = 6. Models were trained using a MSE loss on the energy with the Adam optimizer^69^ in PyTorch^57^, with default parameters of *β*_1_ = 0.9, *β*_2_ = 0.999, and *ϵ* = 10^−8^ without weight decay. In addition, we use gradient clipping by norm with a maximum norm of 100. The dataset was re-shuffled after each epoch. We used a learning rate of 0.001 and a batch size of 16. The learning rate was reduced using an on-plateau scheduler based on the validation MAE of the energy with a patience of 25 and a decay factor of 0.8. We use an exponential moving average with weight 0.999 to evaluate on the validation set as well as for the final model. Training was stopped when one of the following conditions was reached: (a) a maximum training time of approximately 14 days, (b) a maximum number of epochs of 100,000, (c) no improvement in the validation loss for 1000 epochs, (d) the learning rate dropped lower than 1e-5. All models were trained with float32 precision. Again, we note that such long wall times are not required to obtain highly accurate models. We subtract the sum of the reference atomic energies and then apply the linear fitting procedure described above using every 100th reference label in the training set.

### Scaling experiments

Scalability across devices is achieved by implementing an Allegro extension to the LAMMPS molecular dynamics code^66^. The local nature of the Allegro model is compatible with the spatial decomposition approach used in LAMMPS and thus all communication between MPI ranks is handled by existing LAMMPS functionality. The Allegro extension simply transforms the LAMMPS neighbor lists into the format required by the Allegro PyTorch model and stores the resulting forces and energies in the LAMMPS data structures. These operations are performed on the GPU and use the Kokkos performance portability library^72^ to entirely avoid expensive CPU work or CPU-GPU data transfer. The scaling experiments were performed on NVIDIA DGX A100s on the ThetaGPU cluster at the Argonne Leadership Computing Facility, where each node contains 8 GPUs and a total of 320 GB of GPU memory. For the Li_3_PO_4_ simulation, we use a time step of 2 fs, identical to the reference AIMD simulations, float32 precision, and a temperature of *T* = 600 K on the quenched structure, identical to the production simulations used in the quench simulation. For Ag, we use a time step of 5 fs, a temperature of *T* = 300 K and again float32 precision. Simulations were performed for 1000 time steps after initial warm-up.

### Atom-density representations

The Atomic Cluster Expansion (ACE) is a systematic scheme for representing local atomic environments in a body-ordered expansion. The coefficients of the expansion of a particular atomic environment serve as an invariant description of that environment. To expand a local atomic environment, the local atomic density is first projected onto a combination of radial basis functions *R* and spherical harmonic angular basis functions $\overrightarrow{Y}$:

31 $${A}_{zn\ell }=\mathop{\sum}\limits_{j\in {{{{{{{\mathcal{N}}}}}}}}(i)\,{{\mbox{s.t.}}}\,{z}_{j}=z}{R}_{n\ell }({r}_{ij}){\overrightarrow{Y}}_{\ell }^{m}({\hat{r}}_{ij})$$

where *z* runs over all atom species in the system, *z*_*j*_ is the species of atom *j*, ${{{{{{{\mathcal{N}}}}}}}}(i)$ is the set of all atoms within the cutoff distance of atom *i*, also known as its “neighborhood”, and the *n* index runs over the radial basis functions. The *m* index on *A* is implicit. The basis projection of body order *ν* + 1 is then defined as:

32 $${B}_{\begin{array}{c}{z}_{1},{n}_{1}\end{array}}^{(\nu=1)}={A}_{{z}_{1}{n}_{1}{\ell }_{1}=0}$$

33 $${B}_{\begin{array}{c}{z}_{1},{z}_{2},{n}_{1},{n}_{2}\\ {\ell }_{1},{\ell }_{2}\end{array}}^{(\nu=2)}={A}_{{z}_{1}{n}_{1}{\ell }_{1}}\otimes {A}_{{z}_{2}{n}_{2}{\ell }_{2}}$$

34 $$...$$

35 $${B}_{\begin{array}{c}{z}_{1}...{z}_{\nu },{n}_{1}...{n}_{\nu }\\ {\ell }_{1}...{\ell }_{\nu }\end{array}}^{(\nu )}=\mathop{\bigotimes}\limits_{\alpha=1,...,\nu }{A}_{{z}_{\alpha }{n}_{\alpha }{\ell }_{\alpha }}.$$

Only tensor products outputting scalars—which are invariant, like the final target total energy—are retained here. For example, in Eq. (33), only tensor products combining basis functions inhabiting the same rotation order *ℓ*_1_ = *ℓ*_2_ can produce scalar outputs. The final energy is then fit as a linear model over all the scalars *B* up to some chosen maximum body order *ν* + 1.

It is apparent from Eq. (35) that a core bottleneck in the Atomic Cluster Expansion is the polynomial scaling of the computational cost of evaluating the *B* terms with respect to the total number of two-body radial–chemical basis functions *N*_full-basis_ as the body order *ν* + 1 increases: ${{{{{{{\mathcal{O}}}}}}}}({N}_{{{{{{{{\rm{full-basis}}}}}}}}}^{\nu })$. In the basic ACE descriptor given above, *N*_full-basis_ = *N*_basis_ × *S* is the number of radial basis functions times the number of species. Species embeddings have been proposed for ACE to remove the direct dependence on *S*^73^. It retains, however, the ${{{{{{{\mathcal{O}}}}}}}}({N}_{{{{{{{{\rm{full-basis}}}}}}}}}^{\nu })$ scaling in the dimension of the embedded basis *N*_full-basis_. NequIP and some other existing equivariant neural networks avert this unfavorable scaling by only computing tensor products of a more limited set of combinations of input tensors. The NICE framework^74^ is an idea closely related to ACE that aims to solve the problem of increasing numbers of features by selecting only certain features at each iteration based on principal component analysis.

### Normalization

#### Internal normalization

The normalization of neural networks’ internal features is known to be of great importance to training. In this work we follow the normalization scheme of the e3nn framework^75^, in which the initial weight distributions and normalization constants are chosen so that all components of the network produce outputs that element-wise have approximately zero mean and unit variance. In particular, all sums over multiple features are normalized by dividing by the square root of the number of terms in the sum, which follows from the simplifying assumption that the terms are uncorrelated and thus that their variances add. Two consequences of this scheme that merit explicit mention are the normalization of the embedded environment and atomic energy. Both the embedded environment (Eq. (4)) and atomic energy (Eq. (6)) are sums over all neighbors of a central atom. Thus we divide both by $\sqrt{\langle|{{{{{{{\mathcal{N}}}}}}}}(i)|\rangle }$ where $\langle|{{{{{{{\mathcal{N}}}}}}}}(i)|\rangle$ is the average number of neighbors over all environments in the entire training dataset.

#### Normalization of targets

We found the normalization of the targets, or equivalently the choice of final scale and shift parameters for the network’s predictions (see Eq. (5)), to be of high importance. For systems of fixed chemical composition, our default initialization is the following: *μ*_*Z*_ is set for all species *Z* to the average per-atom potential energy over all training frames $\left\langle \frac{{E}_{{{{{{{{\rm{config}}}}}}}}}}{N}\right\rangle$; *σ*_*Z*_ is set for all species *Z* to the root-mean-square of the components of the forces on all atoms in the training dataset. This scheme ensures size extensivity of the potential energy, which is required if one wants to evaluate the potential on systems of different size than what it was trained on. We note that the widely used normalization scheme of subtracting the mean total potential energy across the training set violates size extensivity.

For systems with varying chemical composition, we found it helpful to normalize the targets using a linear pre-fitting scheme that explicitly takes into account the varying chemical compositions: *μ*_*Z*_ is computed by ${[{N}_{{{{{{{{\rm{config}}}}}}}},Z}]}^{-1}[{E}_{{{{{{{{\rm{config}}}}}}}}}]$, where $[{N}_{{{{{{{{\rm{config}}}}}}}},Z}]$ is a matrix containing the number of atoms of each species in the reference structures, and $[{E}_{{{{{{{{\rm{config}}}}}}}}}]$ is a vector of reference energies. Details of the normalization calculations and the comparison between different schemes can be found in ref. ^68^.

### Reporting summary

Further information on research design is available in the Nature Portfolio Reporting Summary linked to this article.

## Supplementary information

Supplementary Information

Reporting Summary

## Data Availability

The Li_3_PO_4_ and Ag data generated in this study have been deposited in the MaterialsCloud database at https://archive.materialscloud.org/record/2022.128. The revMD-17, 3BPA, and QM9 datasets are publicly available (see “Methods”).

## Code availability

An open-source software implementation of Allegro is available at https://github.com/mir-group/allegro together with an implementation of the LAMMPS software interface at https://github.com/mir-group/pair_allegro.
