# Supplementary material for: Learning local equivariant representations for large-scale atomistic dynamics
Source: Nat Commun. 2023 Feb 3;14:579. doi: 10.1038/s41467-023-36329-y (PMC9898554; doi:10.1038/s41467-023-36329-y)
Supplement: Supplementary file 1 — Supplementary Information [file 41467_2023_36329_MOESM1_ESM.pdf]

Supplementary Information

Learning Local Equivariant Representations for Large-Scale Atomistic Dynamics

A. Musaelian, S. Batzner et al.

Supplementary Note 1

As discussed in the section on normalization, the components of our networks are designed and normalized to produce outputs of zero mean and unit variance given inputs with the same. The radial Bessel basis we use (equation 8, 14) does not, as defined, satisfy this property. We found it beneficial to numerically shift and scale the basis functions to make them more closely satisfy this property. In particular we define

$$\mu_n = \int_{r=0}^{r_{\max}} e_{\mathrm{RBF},n} \mathrm{d}r \tag{1}$$

$$\sigma_n^2 = \int_{r=0}^{r_{\max}} (e_{\mathrm{RBF},n} - \mu_n)^2 \mathrm{d}r \tag{2}$$

$$B_n(r_{ij}) = u(r) \frac{e_{\mathrm{RBF},n} - \mu_n}{\sigma_n} \tag{3}$$

where $e_{\mathrm{RBF},n}$ is the nth radial Bessel basis function and $u(r)$ the smooth polynomial cutoff from [1]. These equations follow from assuming a simple uniform distribution of r between 0 and $r_{\max}$.

Supplementary Note 2

The residual update at layer L is computed as a weighted sum:

$$\mathbf{x}^{ij,L} = \frac{1}{\sqrt{1+\alpha^2}} \mathbf{x}^{ij,L-1} + \frac{\alpha}{\sqrt{1+\alpha^2}} \mathbf{x}^{ij,L}. \tag{4}$$

where $\alpha = \frac{1}{2}$ is a hyperparameter. The forms of the coefficients in equation 4 are chosen to enforce normalization: assuming that at initialization $\mathbf{x}^{ij,L-1}$ and $\mathbf{x}^{ij,L}$ are negligibly correlated and each have approximately unit variance and zero mean, the residual sum will then also have approximately unit variance and zero mean.

Supplementary Tables

Table 1 shows an ablation of the local cutoff r_c for the naphthalene molecule in the revised MD17 data-set.

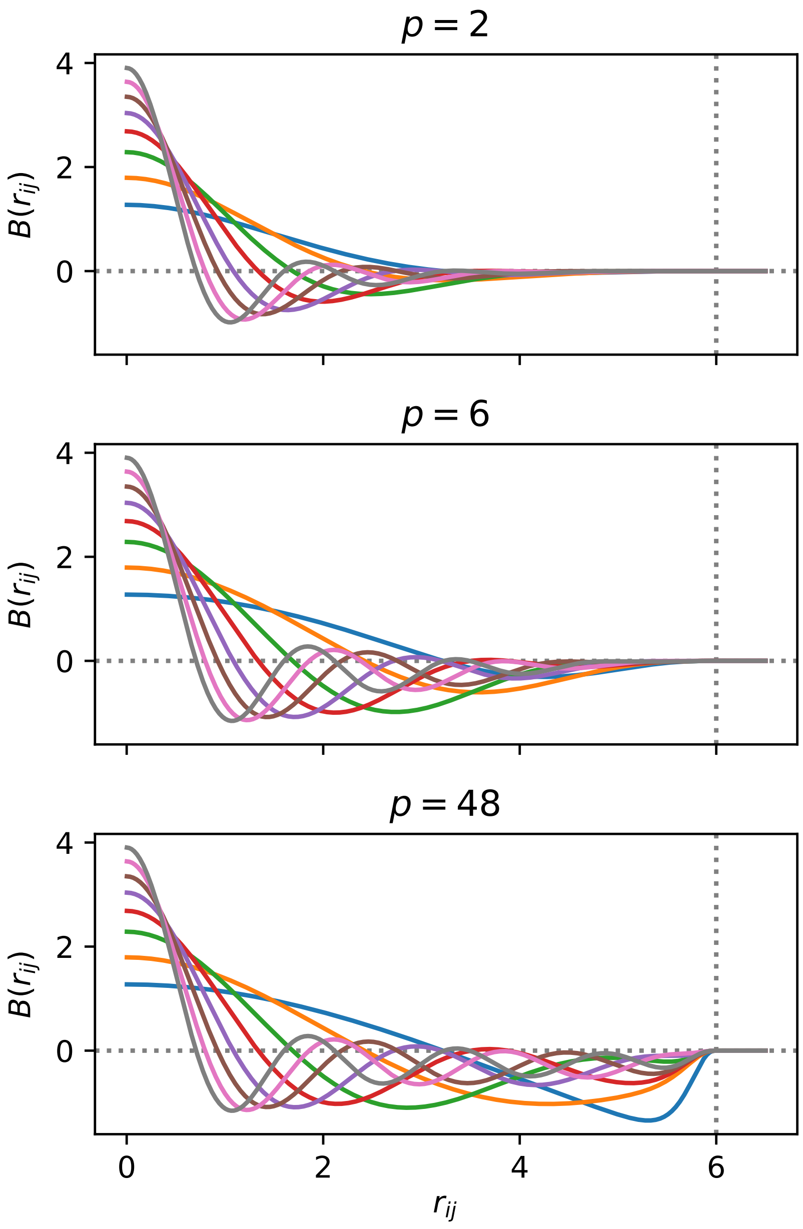

Supplementary Figure 1: The normalized two-body radial basis functions $B_n(r_{ij})$ for $N_{\text{basis}} = 8$. The three panels show different values for the parameter p of the smooth cutoff envelope. The cutoff r_{max} is marked by the vertical line.

Cutoff in Å		Allegro
7	*Energy*	0.5
	Forces	0.9
8	*Energy*	0.5
	Forces	0.9
9	*Energy*	0.5
	Forces	0.9
10	*Energy*	0.5
	Forces	0.9
11	*Energy*	0.5
	Forces	0.9
12	*Energy*	0.5
	Forces	0.9
13	*Energy*	0.5
	Forces	0.9
14	*Energy*	0.5
	Forces	0.9

Supplementary Table 1: Results for the naphthalene molecules in the revised MD17 data set as a function of cutoff.

Supplementary References

[1] Klicpera, J., Groß, J. & Günnemann, S. Directional message passing for molecular graphs. *arXiv preprint arXiv:2003.03123* (2020).
